# Transcription factor TCF7L1 targeting HSPB6 is involved in EMT and PI3K/AKT/mTOR pathways in bladder cancer

**DOI:** 10.1016/j.jbc.2024.108024

**Published:** 2024-11-26

**Authors:** Zizhi Li, Junyi Li, Qingfei Cao, Tong Shen, Yingjie Wang, Haoyang He, Ming Tong

**Affiliations:** 1 Department of Medicine, Soochow University, Soochow, Jiangsu, China; 2 Department of Urology, The First Affiliated Hospital of Jinzhou Medical University, Jinzhou, Liaoning, China

**Keywords:** bladder cancer, heat shock protein B6, transcription factor 7-like 1, progression, EMT, PI3K/AKT/mTOR

## Abstract

Bladder cancer is notorious for its high recurrence and costly treatment burden, prompting a search for novel therapeutic targets. Our study focuses on HSPB6, a small heat shock protein whose reduced expression in bladder cancer suggests a role in tumor biology. Using an integrative approach of bioinformatics, RNA sequencing, and cell-based assays, we show that HSPB6 upregulation inhibits cancer cell proliferation and metastasis while promoting apoptosis. Moreover, TCF7L1-mediated upregulation of HSPB6 leads to suppression of the PI3K/AKT/mTOR signaling pathway, a key driver of cancer progression. These results position HSPB6 as a compelling target for bladder cancer therapy, and its regulatory role in the PI3K/AKT/mTOR axis underscores its therapeutic potential. Our findings pave the way for future investigations into HSPB6-centered treatment strategies.

Globally, bladder cancer (BLCA) is recognized as a significant health threat and ranks as one of the top cancers. The GLOBOCAN 2020 statistics rank BLCA as the world's 10th most prevalent cancer, with yearly estimates indicating 573,000 new cases and 213,000 fatalities. Notably, BLCA exhibits a higher incidence and mortality rate in men, where it ranks as the sixth leading cause of cancer-related deaths and occurrences, with male rates being roughly four times that of females (1). Urothelial carcinoma emerges as the most common BLCA subtype, notorious for its recurrent nature. BLCA is divided into non-muscle invasive bladder cancer (NMIBC) and muscle-invasive bladder cancer (MIBC) based on tumor invasion depth, with MIBC associated with an elevated risk of recurrence, metastasis, and, therefore, a poorer outcome (2).

The primary methods for diagnosing BLCA include cystoscopy and biopsy analysis. Magnetic resonance imaging (MRI) is integral for tumor staging, playing a vital role in determining the most appropriate therapeutic strategies. In the case of NMIBC or low-grade urothelial carcinoma, the conventional treatment regimen involves transurethral resection of the bladder tumor (TURBT). This procedure involves removing the tumor and a margin of healthy tissue surrounding it. Following TURBT, additional treatments may be applied, including *Bacillus* Calmette-Guérin (BCG) therapy or direct chemotherapy instillation into the bladder (2, 3). In cases of MIBC or recurrent NMIBC, the recommendation shifts to radical cystectomy with urinary diversion, complemented by options such as neoadjuvant chemotherapy, immunotherapy, targeted therapy, and radiotherapy to enhance patient survival prospects (4).

In recent years, with the emergence of novel molecular marker detection technologies for BLCA, along with the clinical application of new immunotherapeutic agents and targeted therapies, including Antibody-Drug Conjugates (ADCs), there has been a noticeable reduction in BLCA mortality rates. However, approximately 44% of NMIBC cases still experience local recurrence or progress to MIBC (5, 6, 7, 8). Moreover, the treatment duration and cost for BLCA remain relatively high in the field of oncology. Therefore, developing more efficient, precise, and cost-effective molecular diagnostic and therapeutic approaches is of significant clinical importance in the management of BLCA.

Heat shock proteins (HSPs), crucial molecular chaperones, are ubiquitously present across normal and cancerous human tissues, characterized by their highly conserved molecular architecture. These proteins are essential for ensuring the stability and functionality of cellular proteins. HSPs are classified into different families based on their molecular weights, including HSPH (HSP110), HSPC (HSP90), HSPA (HSP70), DNAJ (HSP40), and HSPB. The HSPB family encompasses the small heat shock proteins (9). Among them, Heat shock protein B6 (HSPB6), part of the small HSP family, weighs in at 17 kDa and is recognized for its distinctive α-crystallin domain, first discovered in skeletal muscle (10). HSPB6 exhibits widespread expression in a variety of tissues, with notable concentrations in cardiac, smooth, and skeletal muscles. Initial research linked HSPB6 to a variety of physiological responses, including vascular and bronchial relaxation, platelet functionality, insulin sensitivity, asthma, atherosclerosis, and wound repair (11, 12). The investigation into HSPB6's involvement in cancer has been expanding, with a 2007 study identifying its presence in hepatocellular carcinoma (HCC) tissues, followed by further research into its role in HCC (13, 14). By 2015, its reduced expression was noted in colorectal cancer by Ju *et al.*, and in 2022, Yang *et al.* explored HSPB6's inhibitory effect on breast cancer development (15, 16). Although the function of HSPB6 in BLCA remains to be fully elucidated, recent bioinformatics research using databases like GEO and TCGA has begun to integrate HSPB6 into prognostic models to probe its potential role in BLCA's development and advancement (17, 18, 19, 20).

TCF7L1, a constituent of the TCF/lymphoid enhancer factor (LEF) family, assumes a pivotal function in the preservation of stem cell pluripotency (21). Studies indicate that TCF7L1 plays a crucial role in attaching to TCF consensus sequences within DNA, collaborating with fellow TCF family members—TCF7, LEF1, and TCF7L2—to jointly influence the gene expression governed by the Wnt/β-catenin signaling cascade (22). The influence of TCF7L1 has been detected in several types of cancer, such as skin squamous cell carcinoma, colorectal, gastric, and prostate cancers, underscoring its pivotal contribution to the mechanisms of cancer development (23, 24, 25).

This research marks the inaugural examination of HSPB6's function in BLCA, delving into the intricate relationship between HSPB6 and TCF7L1 and their collective influence on BLCA progression. The insights gleaned from this study illuminate novel therapeutic avenues for BLCA treatment, providing a foundation for future interventions aimed at combating this malignancy.

## Results

### Differential expression of HSPB6 across various tumors and in BLCA

**Figure 1:**
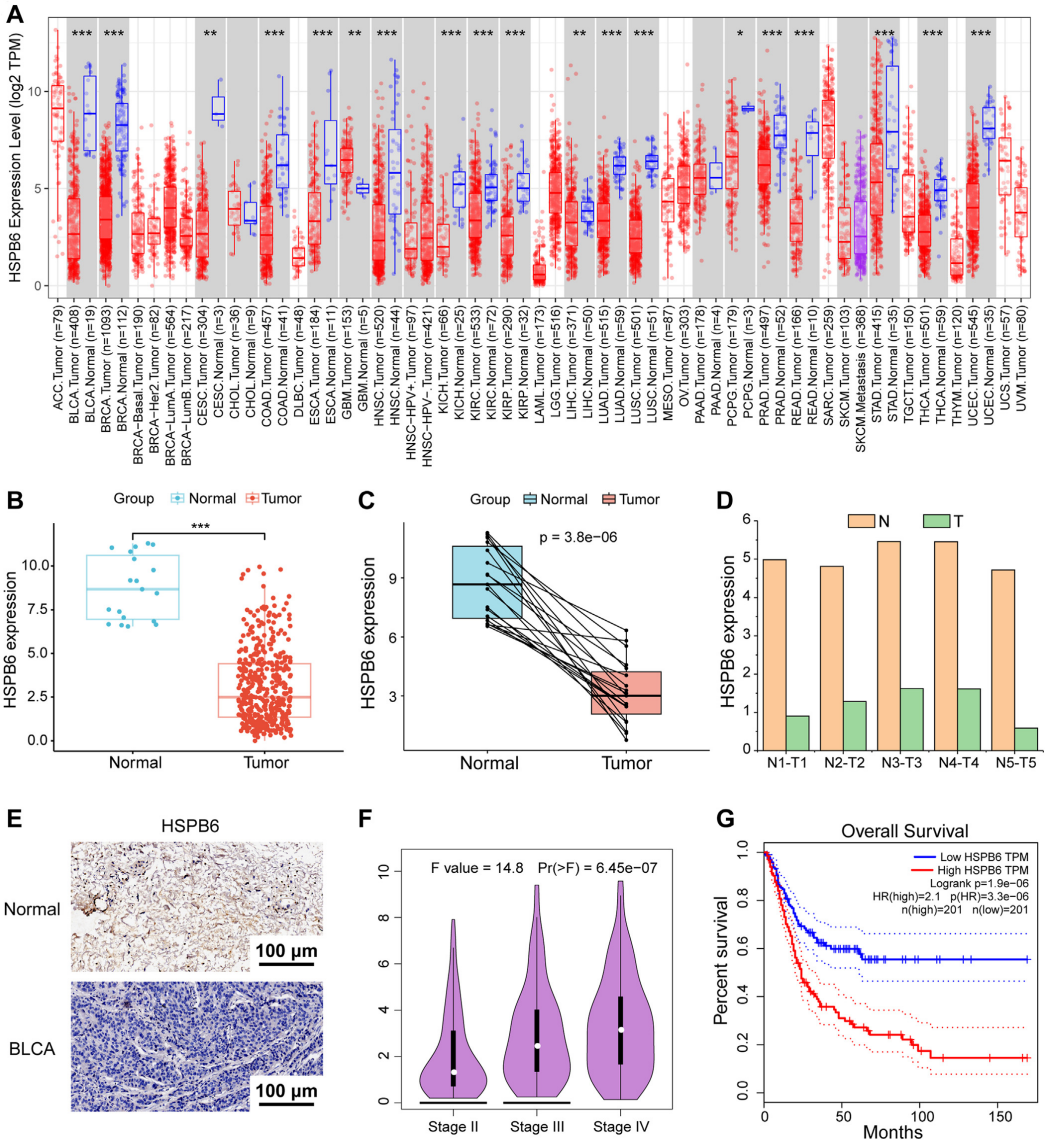
**The differential expression of HSPB6 in several tumors and BLCA.***A*, the mRNA expression of HSPB6 in several tumors in the GEPIA2 database. *B* and *C*, the mRNA expression of HSPB6 in BLCA tissues and corresponding normal tissues in TCGA database (*p* < 0.001). *D*, the expression of HSPB6 of five paired cancer tissue and paracancerous tissue specimens in our hospital. *E*, IHC investigate HSPB6 expression in BLCA and normal tissues. *F*, HSPB6 expression in tumor stage was analyzed using the GEPIA2 database. *G*, Kaplan-Meier analysis indicated the predictive significance of low HSPB6 expression vs high HSPB6 expression in patients with BLCA in the GEPIA2 database (*p* < 0.001). ∗∗∗*p* < 0.001; ∗∗*p* < 0.01; ∗*p* < 0.05.

Utilizing pan-cancer sequencing data from the TCGA database, we explored the expression levels of HSPB6 across a spectrum of tumor types and their corresponding normal tissues. Our study revealed a consistent decrease in HSPB6 expression across various cancers, including BLCA, breast carcinoma, and cervical squamous cell carcinoma, among others (Fig. 1, *A*–*C*). The observed decrease in HSPB6 expression pattern was further validated in paired cancerous and adjacent non-cancerous tissue samples obtained from patients undergoing radical cystectomy at our institution, thus confirming its diminished presence in cancer tissues (Fig. 1*D*). IHC further corroborated the decreased expression of HSPB6 specifically in BLCA tissues (Fig. 1*E*). Interestingly, upon assessing HSPB6 expression across different stages of BLCA, an increase was observed in Stage IV samples relative to Stages II & III (Fig. 1*F*). Moreover, survival analysis indicated that elevated HSPB6 levels are linked to increased mortality in patients with BLCA (Fig. 1*G*).

### Impact of HSPB6 overexpression on BLCA cell proliferation and apoptosis

**Figure 2:**
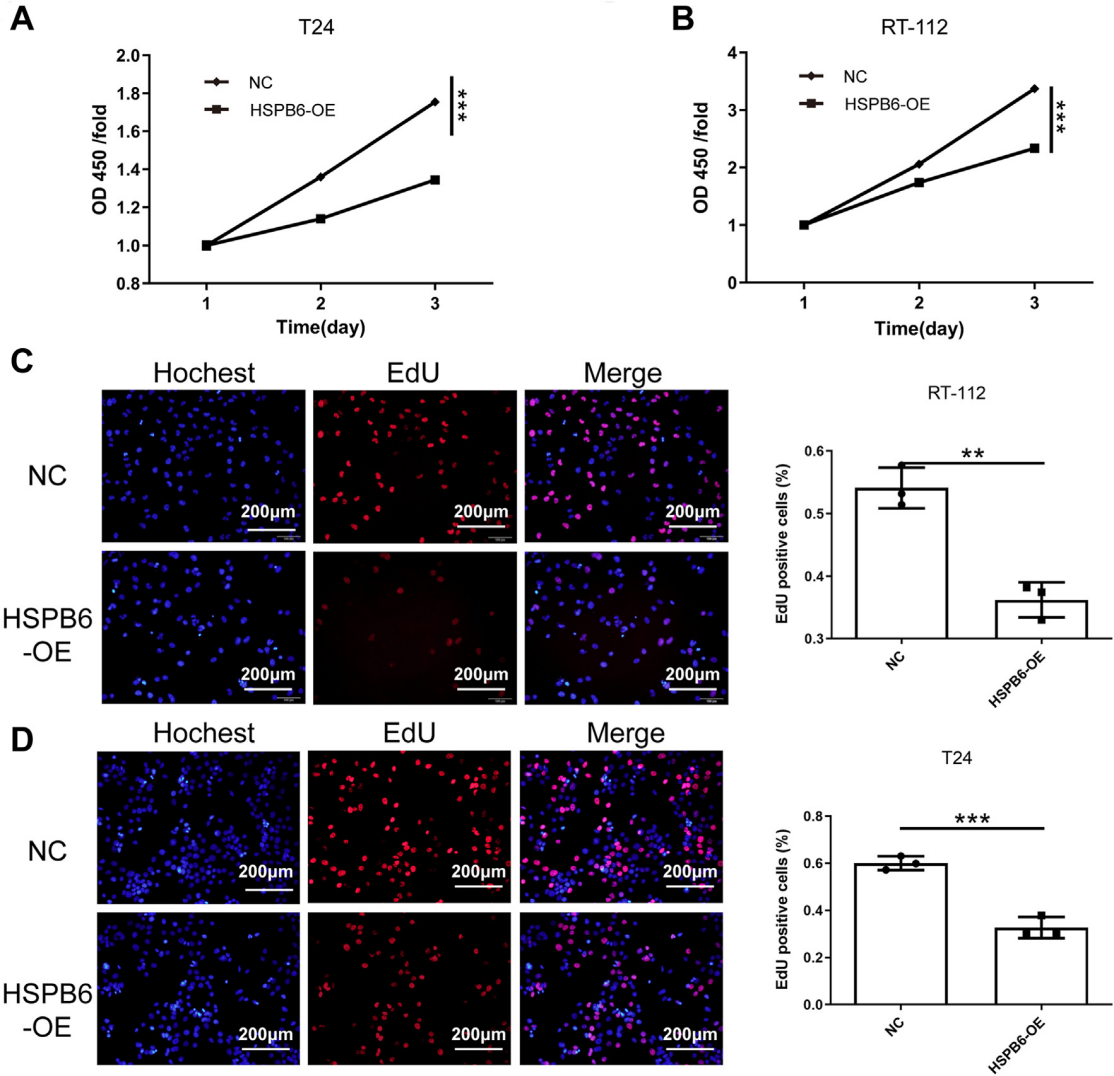
**The overexpression of HSPB6 inhibits the proliferation in bladder cancer cells.***A* and *B*, T24 and RT-112 cells viability were evaluated using MTT assay. *C* and *D*, EdU assays indicated that HSPB6 overexpression impacted the proliferation of T24 and RT-112 cells. ∗∗∗*p* < 0.001; ∗∗*p* < 0.01; ∗*p* < 0.05.

**Figure 3:**
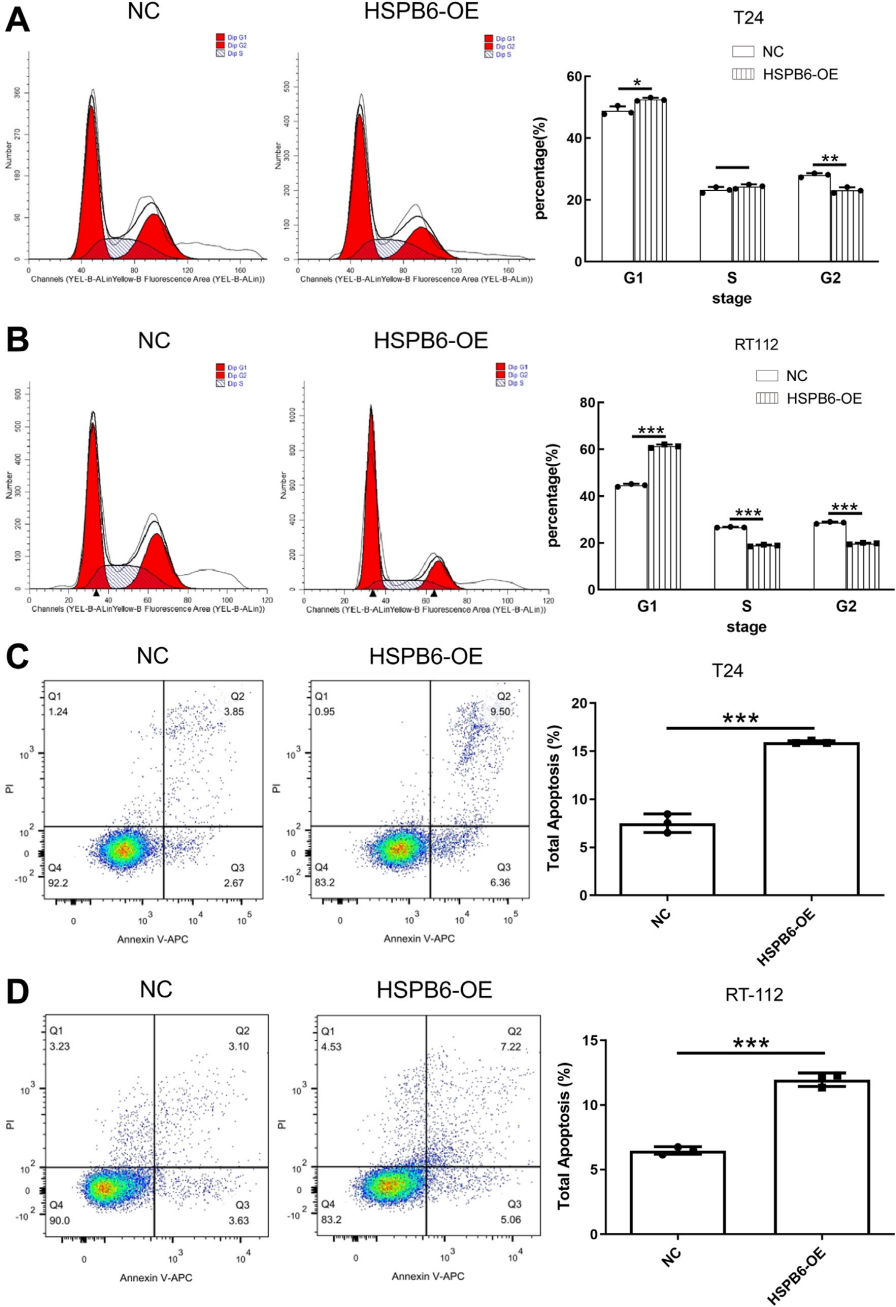
**The overexpression of HSPB6 promotes apoptosis in bladder cancer cells.***A* and *B*, flow cytometry analysis of the cell cycle and statistical results of T24 and RT-112 cells with HSPB6 overexpression were revealed. *C* and *D*, flow cytometry was performed for the detection of the apoptosis of T24 and RT-112 cells. ∗∗∗*p* < 0.001; ∗∗*p* < 0.01; ∗*p* < 0.05.

In our investigation into HSPB6's role within BLCA, T24 and RT-112 cells were selected due to their notably reduced HSPB6 expression compared to normal bladder cell line HCV-29, and a more marked expression disparity relative to other BLCA lines (5637, HT-1376) (Fig. S1*A*). Addressing HSPB6's scarce presence in BLCA, we employed an overexpression lentivirus from Shanghai Jikai Gene Chemical Technology Co., Ltd to achieve stable HSPB6 upregulation in BLCA cells, verified in Fig. S1, *B* and *C*. Subsequent CCK-8 assays demonstrated that HSPB6 elevation significantly curtailed T24 and RT-112 cell proliferation (Fig. 2, *A* and *B*). EdU proliferation assays corroborated this finding, illustrating a decline in cell proliferation following HSPB6 augmentation (Fig. 2, *C* and *D*). Further analysis of apoptosis revealed that HSPB6 overexpression markedly enhanced apoptotic rates in both T24 and RT-112 cells (Fig. 3, *A* and *B*). Moreover, cell cycle distribution analyses indicated a pronounced G1 phase arrest in cells with HSPB6 overexpression compared to the negative control group (Fig. 3, *C* and *D*), underscoring HSPB6's pivotal role in modulating BLCA cell cycle progression and survival.

### Suppression of BLCA cell migration and invasion through HSPB6 upregulation

**Figure 4:**
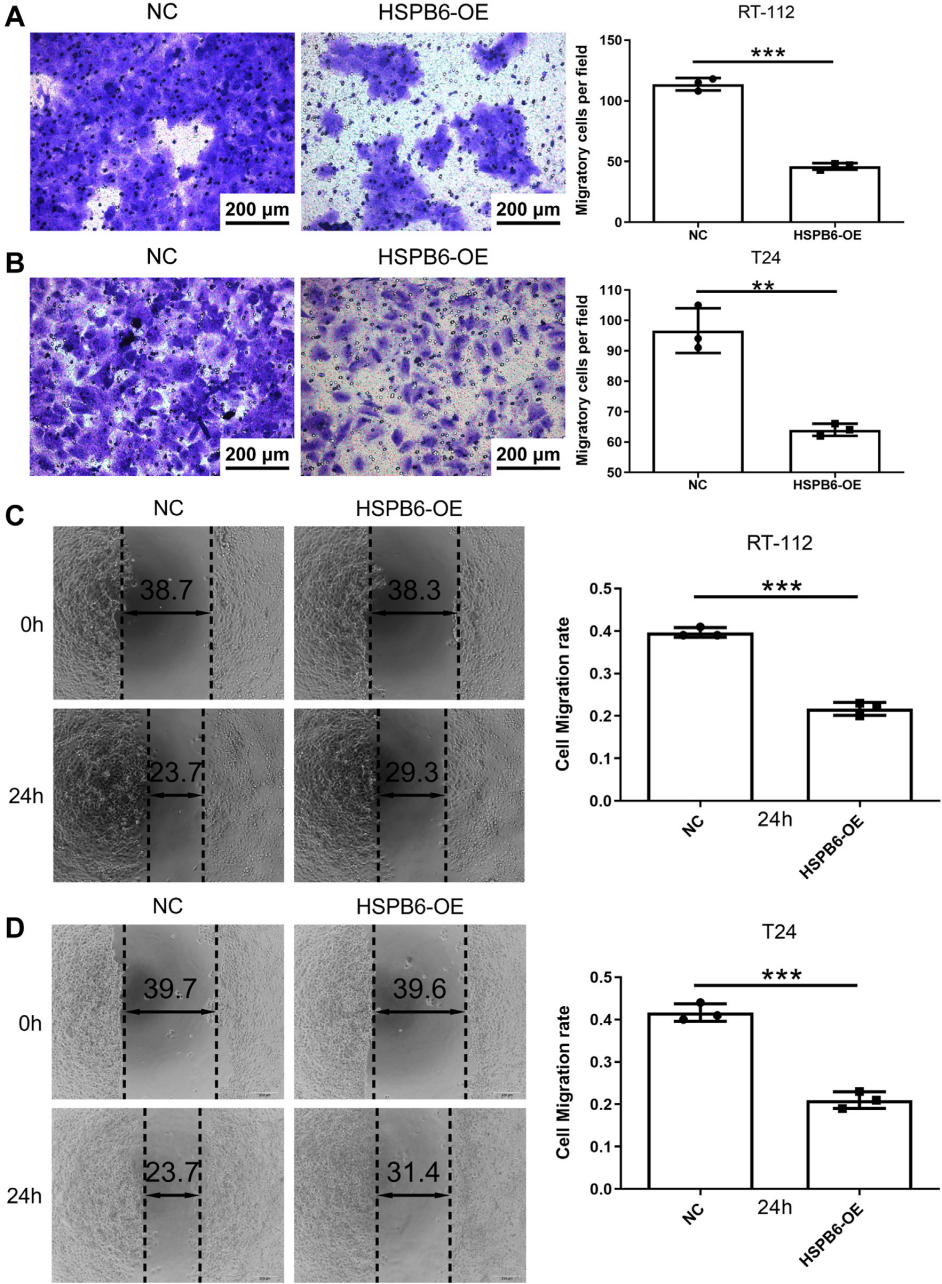
**HSPB6 overexpression suppressed bladder tumor cell migration and invasion.***A* and *B*, transwell assays and quantification of cell migration rate was performed to examine T24 and RT-112 cell migration, respectively. *C* and *D*, wound healing and Quantification of cell invasion rate were performed to examine T24 and RT-112 cell migration, respectively. ∗∗∗*p* < 0.001; ∗∗*p* < 0.01.

Recognizing the critical importance of cell migration in the advancement of cancer (26), we initiated a comprehensive set of experiments to assess the migratory and invasive behaviors of bladder cancer cells. Through the use of Transwell assays, a marked diminishment in the invasive capacities of T24 and RT-112 cells was noted following the overexpression of HSPB6 (Fig. 4, *A* and *B*). This diminished capacity for the invasion was corroborated by results from wound healing assays, indicating a significant suppression of migratory activity in both cell lines with the upregulation of HSPB6 (Fig. 4, *C* and *D*). These results collectively highlight the strong suppressive impact of HSPB6 elevation on the migration and invasion activities of bladder cancer cells, pointing to its potential value as a therapeutic target in halting the progression of bladder cancer.

### Effect of HSPB6 overexpression on bladder carcinoma growth in vivo

**Figure 5:**
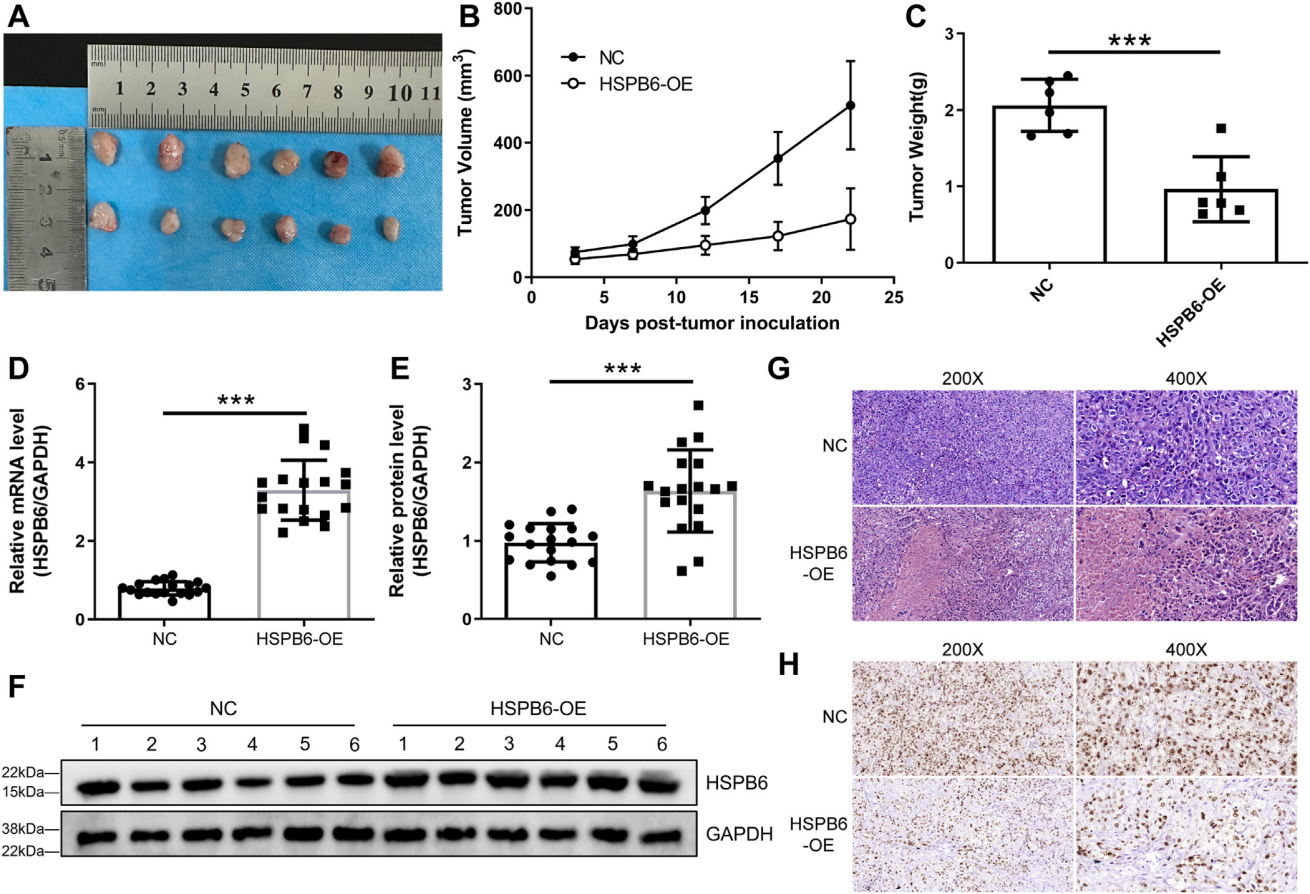
**HSPB6 overexpression suppressed bladder carcinoma growth *in vivo*.***A*, male BALB/c nude mice were subcutaneously injected with T24 cells transfected with NC or HSPB6-OE. After sacrifice, the tumors removed from each mouse were photographed. *B*, tumor size was measured at days 3, 7, 12, 17 and 22 after inoculation. *C*, tumor weight was recorded. *D*, the mRNA expression of HSPB6 in tumor tissues of mice was examined using RT-qPCR. *E* and *F*, the protein expression of HSPB6 in tumor tissues of mice was examined using Western blot analysis. *G* and *H*, HE and Ki-67 staining elucidated the variations in the NC and HSPB6 overexpression groups. ∗∗∗*p* < 0.001.

In our investigation of HSPB6's influence on the growth of bladder carcinoma within a live setting, we utilized a xenograft tumor model in male BALB/c nude mice. These mice received subcutaneous injections of T24 cells, which were previously transfected with either a non-coding (NC) vector or an HSPB6 overexpression (HSPB6-OE) vector. We systematically monitored tumor growth, documenting its progression on days 3, 7, 12, 17, and 22 after the cells were injected. According to the results showcased in Figure 5, *A*–*C*, there was a noticeable decline in tumor volume and mass in the mice that received cells with HSPB6 overexpression, illustrating the inhibitory role of HSPB6 in tumor growth. Further examination of the tumors disclosed a significant increase in HSPB6 mRNA and protein expressions in the HSPB6-OE group when compared to the NC group, with statistical significance reaching a *p*-value of less than 0.001 (Fig. 5, *D*–*F*). Additionally, immunohistochemical analyses, including hematoxylin and eosin (HE) and Ki-67 staining, revealed a substantial reduction in expression levels in the tumors with HSPB6 overexpression relative to the control group (Fig. 5, *G* and *H*). These *in vivo* findings collectively emphasize the strong anti-tumor properties of HSPB6 overexpression in combatting bladder carcinoma, suggesting its potential utility as a therapeutic intervention.

### TCF7L1 downregulation in bladder cancer and its regulatory effect on HSPB6

**Figure 6:**
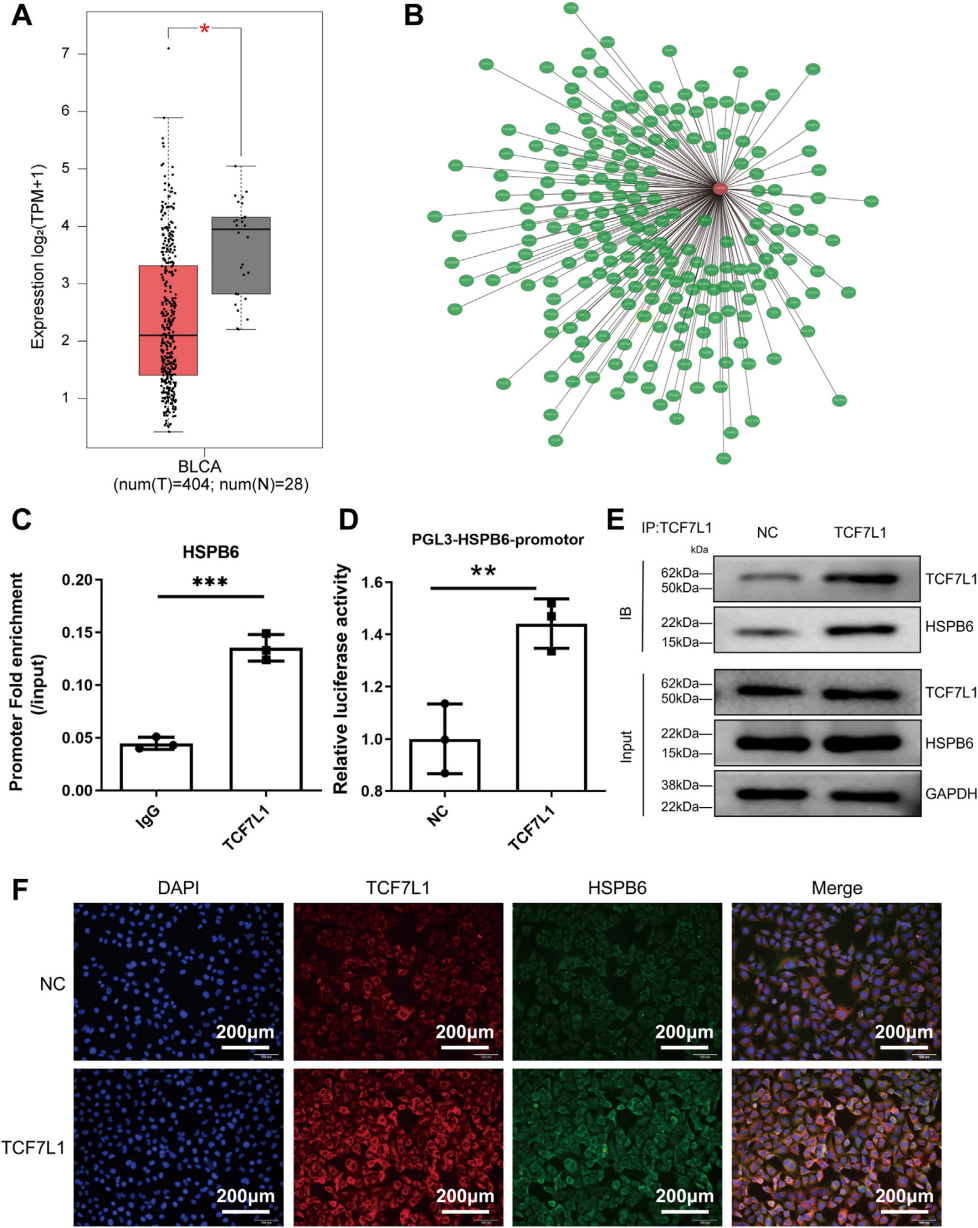
**TCF7L1 expression is downregulated in bladder cancer tissues, inhibiting the transcription and expression of HSPB6 through targeted regulation.***A*, the mRNA expression of HSPB6 in BLCA tissues and normal tissues in GEPIA2 database. *B*, the network of Co-expressed genes with HSPB6 in the Coexpedia database. *C*, T24 cells were transfected with TCF7L1-OE and IgG, and the expression level of HSPB6 was detected using RT-qPCR. *D*, HSPB6 promoter transcriptional activity upon TCF7L1 overexpression was evaluated by luciferase reporter assay. ∗∗*p* < 0.01 vs. NC. *E*, immunoprecipitated chromatin fragments were analyzed using Western blot analysis. *F*, immunofluorescence analysis of TCF7L1 and HSPB6 expression in NC and TCF7L1-OE group in T24 cells. Scale bars, 200 μm. The data originated from three independent experiments. ∗∗∗*p* < 0.001, ∗∗*p* < 0.01.

Exploring the transcriptional regulation of HSPB6, our investigation extended to the expression of TCF7L1 within bladder cancer tissues. Leveraging data from the GEPIA2 database, we noted a significant reduction in TCF7L1 levels in BLCA specimens when juxtaposed with adjacent non-malignant tissues (Fig. 6*A*). Further analysis using the Coexpedia database highlighted an interaction between TCF7L1 and HSPB6, suggesting a regulatory relationship (Fig. 6*B*). To delve deeper into the dynamics between TCF7L1 and HSPB6, we employed CHIP-PCR assays which revealed that TCF7L1 overexpression notably enhances HSPB6 expression, indicating a positive regulatory effect (Fig. 6, *C*–*E*). Complementing these findings, immunofluorescence assays demonstrated a pronounced increase in HSPB6 expression within TCF7L1-overexpressing cell lines, with a predominant nuclear localization observed (Fig. 6*F*). These findings collectively underscore the pivotal role of TCF7L1 in the transcriptional regulation of HSPB6 in BLCA. The downregulation of TCF7L1 in BLCA tissues contributes to diminished HSPB6 expression, suggesting that enhancing TCF7L1 activity could serve as a strategic avenue for upregulating HSPB6, thereby potentially inhibiting cancer progression.

### Impact of HSPB6 knockdown on the inhibitory role of TCF7L1 overexpression in BLCA progression

**Figure 7:**
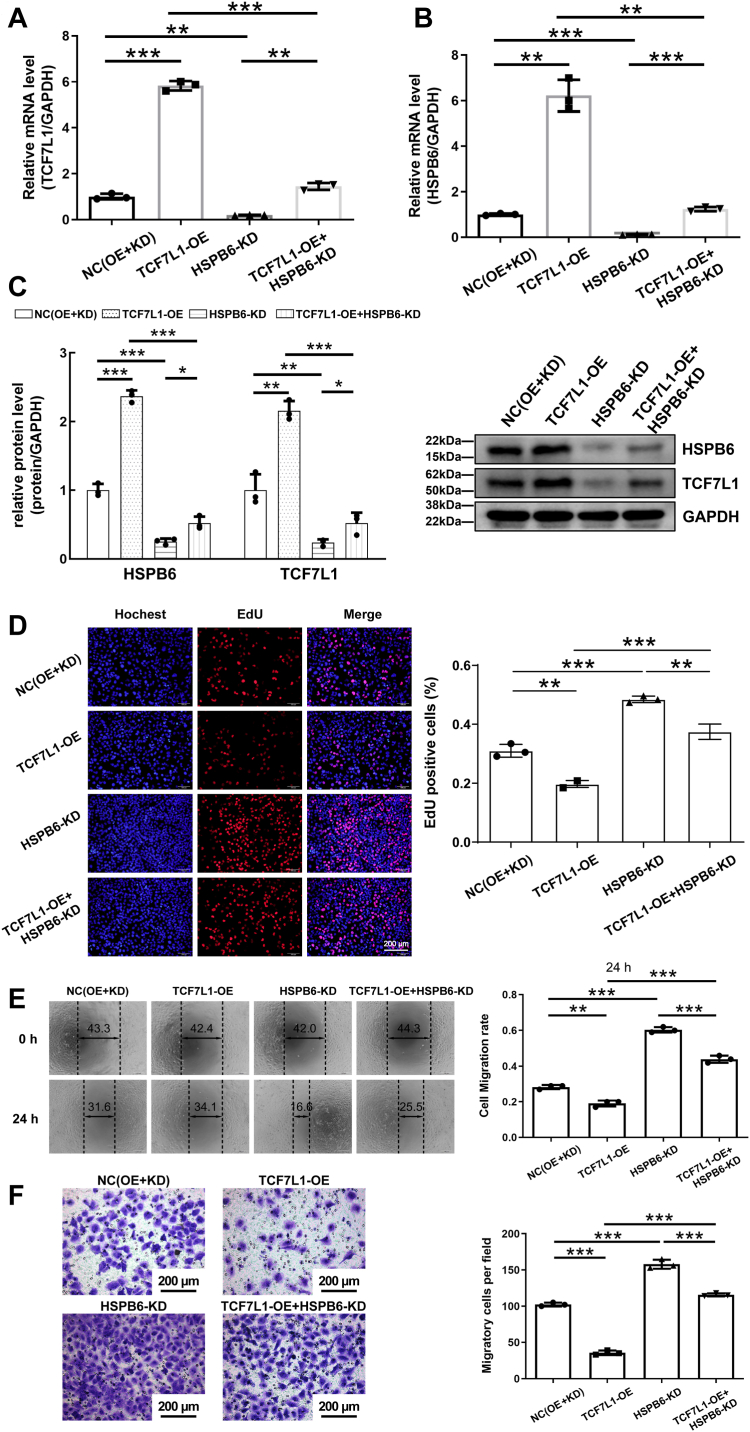
**Knocking down HSPB6 reverses the inhibitory effect of TCF7L1 overexpression on the proliferation and invasion of bladder cancer cells.***A*, the mRNA levels of TCF7L1 in the NC group, TCF7L1 overexpression group, HSPB6 knockdown group, TCF7L1 overexpression + HSPB6 knockdown group in the T24 cell line were detected using RT-qPCR. *B*, the mRNA levels of HSPB6 in four groups were determined using RT-qPCR. *C*, after overexpression of TCF7L1 and simultaneous knockdown of HSPB6, the elevated TCF7L1 and HSPB6 protein expression levels were decreased. ∗∗∗*p* < 0.001 vs. TCF7L1-OE. *D*, EdU assay showed that knocking down HSPB6 reduced the inhibitory effect of overexpressed TCF7L1 on the proliferation of T24 cells. *E* and *F*, Transwell assays and quantification of cell migration rate was performed to examine T24 cell migration, and wound healing and quantification of cell invasion rate were performed to examine T24 cell migration, respectively. ∗∗∗*p* < 0.001; ∗∗*p* < 0.01; ∗*p* < 0.05.

**Figure 8:**
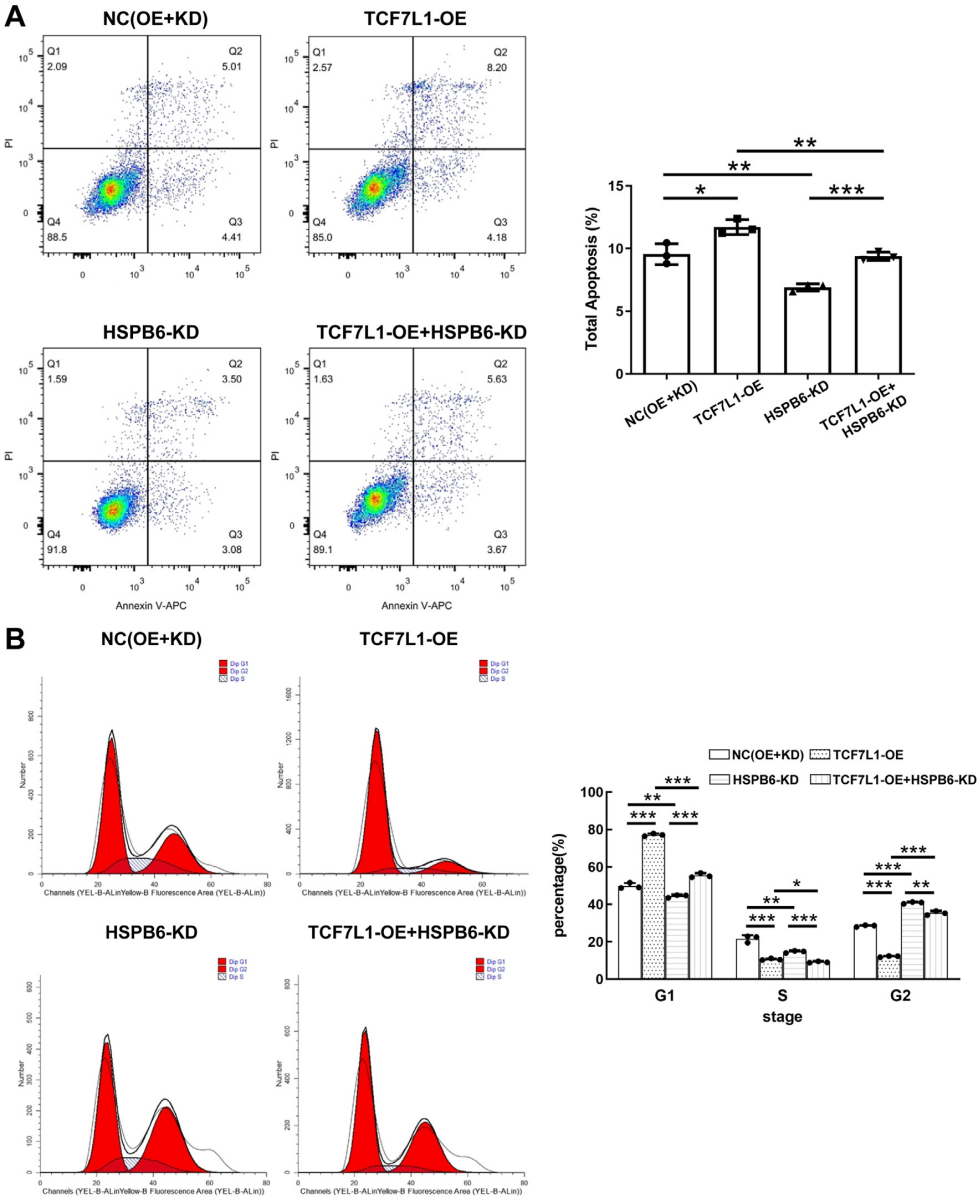
**Knocking down HSPB6 reverses the promoting effect of TCF7L1 overexpression on the apoptosis of bladder cancer cells.***A* and *B*, flow cytometry also indicates that knockdown of HSPB6 reverses the inhibitory effect of overexpressed TCF7L1 on the progression of bladder cancer cells. ∗∗∗*p* < 0.001; ∗∗*p* < 0.01; ∗*p* < 0.05.

Building on the established positive regulation of HSPB6 by TCF7L1, we embarked on rescue experiments to further delineate TCF7L1's functional significance in BLCA. To this end, cells were categorized into four experimental groups: negative control (NC), TCF7L1 overexpression (TCF7L1-OE), HSPB6 knockdown (HSPB6-KD), and a combination of TCF7L1 overexpression with HSPB6 knockdown (TCF7L1-OE + HSPB6-KD). To control the off-target effect, we designed and verified three knockdown sequences of HSPB6 (Fig. S2, *A* and *B*). The efficiency of transfection was confirmed through quantitative RT-PCR and Western blot analyses (Fig. 7, *A*–*C*). EdU assays illustrated that TCF7L1 overexpression diminished tumor cell proliferation, a phenomenon mitigated upon concurrent HSPB6 depletion (Fig. 7*D*). Consistent trends were observed in the evaluation of cell migration and invasion, which were analyzed using wound healing and Transwell assays, respectively, (Fig. 7, *E* and *F*). Additional analyses concerning cell apoptosis and cell cycle dynamics further supported these findings (Fig. 8, *A* and *B*). From these results, it becomes evident that HSPB6 knockdown significantly counteracts the growth-inhibitory and anti-migratory effects imparted by TCF7L1 overexpression in bladder cancer cells. This emphasizes the crucial function of HSPB6 in facilitating the inhibitory effect of TCF7L1 on the progression of BLCA cells, shedding light on the complexities of their regulatory interaction within the cellular environment of bladder cancer.

### HSPB6 overexpression attenuates EMT and dampens the PI3K/AKT/mTOR pathway in BLCA

**Figure 9:**
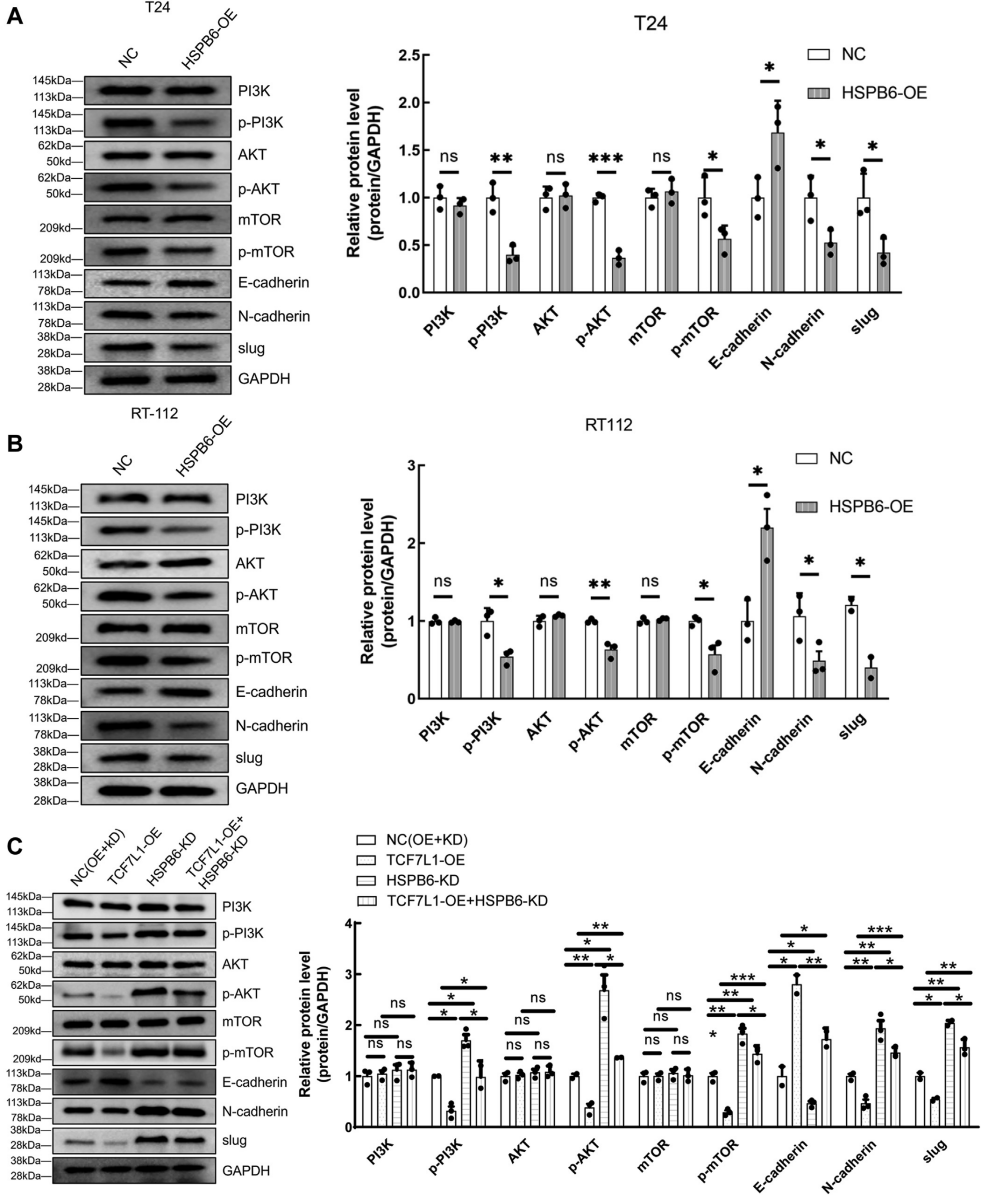
**High expression of HSPB6 inhibits EMT and PI3K/AKT pathway.***A* and *B*, the protein expression of PI3K, p-PI3K, AKT, p-AKT, mTOR, p-mTOR, E-cadherin, N-cadherin, and slug in T24 cell and RT-112 cell were examined using Western blot analysis. *C*, the protein expression of PI3K, p-PI3K, AKT, p-AKT, mTOR, p-mTOR, E-cadherin, N-cadherin and slug in T24 cell was examined in rescue experiment. ∗∗∗*p* < 0.001; ∗∗*p* < 0.01; ∗*p* < 0.05.

The transition of epithelial cells into a mesenchymal state, known as the EMT process, is crucial for boosting the invasiveness and metastatic capabilities of cancer cells (27). In our study of the effects of HSPB6 on BLCA cells, we observed notable changes in the levels of proteins associated with EMT. Overexpressing HSPB6 led to a significant increase in E-cadherin levels, while levels of N-cadherin and Slug proteins saw a decrease in both T24 and RT-112 cells, as shown in Figure 9, *A* and *B*. These findings suggest that the regulatory role of HSPB6 in EMT processes could have a substantial impact on the migration and invasion capacities of BLCA cells.

Recognizing the essential role of the PI3K/AKT/mTOR signaling pathway in tumor growth (28), which influences key cellular functions including survival, proliferation, metabolism, invasion, and angiogenesis, we explored how overexpressing HSPB6 affects this pathway. However, there were no significant differences in the expression levels of PI3K, AKT, and mTOR between the overexpressed group and the control group (Fig. 9, *A* and *B*). In the subsequent rescue experiment, knockdown of HSPB6 down-regulated the expression of E-cadherin in T24 cells overexpressed with TCF7L1 and upregulated the expression levels of p-PI3K, p-AKT, p-mTOR, N-cadherin and slug, PI3K, AKT, and mTOR also showed no significant changes (Fig. 9*C*). These findings reinforce the intricate regulatory interplay between TCF7L1 and HSPB6 concerning EMT and the PI3K/AKT/mTOR pathways. Therefore, HSPB6 serves a critical function not only in regulating EMT but also in suppressing the PI3K/AKT/mTOR signaling pathway, further underscores its potential as a therapeutic target in the treatment of bladder cancer.

## Discussion

BLCA continues to represent a major contributor to cancer-related mortality globally (1). Despite the diverse array of treatment modalities available, such as surgery, intravesical and systemic chemotherapy, immunotherapy, and targeted therapies aimed at initial control of primary tumor growth, the recurrent nature of BLCA and the grim prognosis associated with MIBC remain substantial challenges for oncologists. Therefore, delving into the pathogenesis of BLCA and devising strategies to thwart or mitigate BLCA metastasis is critical.

sHSPs family, which includes 10 molecular chaperones ranging from HSPB1 to HSPB10, is vital for maintaining the stability and functionality of the proteome. Additionally, sHSPs possess anti-apoptotic capabilities by influencing key molecules such as JNK, AKT, and NF-κB (29). Through this modulation, sHSPs impact a variety of diseases, notably cancer, by controlling crucial cellular processes. These include cell proliferation, differentiation, invasion, metastasis, cell death, and how the immune system identifies tumor cells (30). Notably, HSPB1 has been the focus of considerable research within cancer studies, often linked to drug resistance and adverse prognosis (31). Conversely, the role of HSPB6 in BLCA has remained unexplored until this study, which is the first to document the significant underexpression of HSPB6 in BLCA tissue samples and cell lines, unveiling its tumor-suppressive potential. The observed decrease in cell proliferation, migration, and invasion following the overexpression of HSPB6 in T24 and RT-112 cells corroborates this perspective. Additionally, alterations in the expression of key proteins associated with the PI3K/AKT/mTOR and EMT pathways, including p-PI3K, p-AKT, p-mTOR, E-cadherin, N-cadherin, and Slug, further substantiate the regulatory role of HSPB6 in modulating tumor dynamics. *In vivo* experiments complement these findings, demonstrating HSPB6's tumor-suppressive efficacy by reducing tumor growth, thereby highlighting HSPB6 as a promising therapeutic target for BLCA.

EMT is often thought to be related to tumor invasion and metastasis (32). In our study, we detected that after overexpression of HSPB6 and TCF7L1, E-cadherin expression was increased in cell lines, while N-cadherin and Slug expression was decreased. Rescue experiments showed that after overexpression of TCF7L1 and knocking down HSPB6, the expression of these proteins was reversed, indicating that TCF7L1 targeting HSPB6 is involved in the pathological process of EMT in bladder cancer.

PI3K/AKT/mTOR is one of the most common regulatory pathways in malignant tumors, affecting cell proliferation, migration, and metastasis (33). To further investigate whether the PI3K/AKT/mTOR signaling pathway is involved in the carcinogenic mechanism of HSPB6 in BLCA, we evaluated the relationship between HSPB6 and p-PI3K, p-AKT, or p-mTOR. Compared with the control group, the phosphorylation levels of PI3K, AKT, and mTOR were significantly reduced after HSPB6 overexpression, suggesting that HSPB6 inhibited the activation of PI3K/AKT/mTOR signaling pathway in bladder cancer. In the rescue experiment, the knockdown of HSPB6 could partially restore the expression of p-PI3K, p-AKT, or p-mTOR in BLCA by TCF7L1 overexpression, which also indicated the targeted regulatory relationship of TCF7L1 to HSPB6. However, the exact mechanism by which HSPB6 interacts with the PI3K/AKT/mTOR signaling pathway remains to be determined by further studies. *In vivo* experiments further validated HSPB6's tumor-suppressive role, as its overexpression weakened tumor growth. These novel findings suggest that targeting HSPB6 may be a promising strategy for treating BLCA.

Despite the established tumor-suppressing properties of HSPB6 in BLCA, as evidenced through both *in vivo* and *in vitro* studies, bioinformatics analyses have revealed a paradox: HSPB6 expression is notably higher in normal tissues compared to its reduced levels in cancerous counterparts. Notably, stage IV bladder cancers show a higher expression of HSPB6 compared to stages II and III. Furthermore, patients with lower HSPB6 expression levels have significantly better overall survival (OS) rates than those with higher expression levels. This incongruity might be partially explained by the underrepresentation of NMIBC cases within the TCGA database, which, although accounting for about 50 to 70% of bladder cancer cases, points to a potential interpretative bias. Additionally, the complexity of gene regulation networks often eludes prediction based solely on overt clinical characteristics. These findings underscore the multifaceted role of HSPB6 across the cancer spectrum, suggesting that in advanced stages, HSPB6 could potentially shift towards promoting tumorigenesis through mechanisms like angiogenesis enhancement, metastasis facilitation, and apoptosis inhibition (34). To unravel the intricate functions of HSPB6 in cancer more thoroughly, it's imperative to broaden the research framework by incorporating more clinical samples and engaging in deeper investigative pursuits. However, our experiments have solidly demonstrated HSPB6's robust anti-cancer activity in BLCA, reinforcing its potential as a pivotal factor in cancer biology.

Therapeutic strategies targeting transcription factors have demonstrated promising results (35). TCF7L1, a member of the TCF/LEF family, is characterized by a conserved DNA-binding high mobility group box and a β-catenin binding domain located at its amino terminus (9). These features play a vital role in preserving stem cell pluripotency through the Wnt/β-catenin signaling pathway (36). Our study, utilizing the Coexpedia database, identified TCF7L1 as co-expressed with HSPB6 and further validated this interaction through luciferase reporter gene and CHIP assays, indicating TCF7L1's binding to HSPB6's promoter region. Rescue experiments further confirmed TCF7L1's role in inhibiting tumor growth in BLCA, demonstrating that overexpressing TCF7L1 leads to a reduction in cell proliferation, migration, and invasion, alongside an increase in apoptosis within tumor cells. Conversely, knocking down HSPB6 expression partly reversed these effects, suggesting the tumor-suppressive impact of TCF7L1 overexpression is modulated by HSPB6 expression levels. This intricate molecular interplay offers new perspectives for therapeutic strategies in BLCA treatment.

## Conclusion

In summary, this study reveals that the expressions of HSPB6 and TCF7L1 are both downregulated in the tumor tissues of BLCA patients. This finding has been supported by bioinformatics data and research on BLCA cell lines. Upregulating the expression of HSPB6 and TCF7L1 effectively inhibits the proliferation, migration, invasion, and overall growth of BLCA cells, thereby impeding the development of BLCA. At the molecular level, TCF7L1 can bind to the promoter region of HSPB6 and modulate its expression through transcriptional regulation, thereby intervening in the progression of BLCA. This study provides new insights into the potential regulatory mechanisms of BLCA and opens up novel therapeutic target avenues for its treatment.

## Experimental procedures

### Bioinformatics analysis

The bioinformatics analyses of HSPB6 were conducted utilizing the GEPIA2 online platform (http://gepia2.cancer-pku.cn), developed by Zemin Zhang's team at Peking University. This tool is designed to facilitate cancer research by providing a user-friendly interface for gene screening and visualization of expression differences between normal and tumor tissues, both in bladder cancer BLCA and at a pan-cancer level. Utilizing this database, we analyzed gene expression variations and performed Kaplan-Meier survival analyses to prognosticate outcomes for patients with BLCA, anchored on the median expression levels of HSPB6. This methodology underscores the significance of HSPB6 as a potential prognostic marker and its implications in the broader context of cancer biology and therapy.

### Ethical consideration and tissue acquisition

The study received ethical clearance from the Ethics Committee at the First Affiliated Hospital of Jinzhou Medical University and abided by the Declaration of Helsinki principles. A collection of five BLCA specimens alongside their corresponding healthy tissues was secured from patients at the same institution. Before undergoing surgery, these patients had not been subjected to any form of chemotherapy or radiotherapy. All underwent laparoscopic radical cystectomy, with the collected tissues promptly stored in liquid nitrogen post-surgery.

### Cell culture procedures

We acquired cell lines for our study from Guangzhou Cellcook Biotech Co. Ltd in Guangzhou, China. This collection included a normal bladder cell line, HCV-29, alongside various human bladder cancer cell lines: T24, 5637, HT-1376, and RT-112. We cultured these cells using RPMI 1640 medium from Gibco, USA, enriching it with 10% fetal bovine serum from BI, Israel, and adding 1% penicillin/streptomycin, also from Gibco, USA. The culture conditions were meticulously regulated, maintaining a 5% CO2 atmosphere and a steady temperature of 37 °C. To ensure the integrity of our experiments, we rigorously checked all cell lines for *mycoplasma* contamination before beginning any experimental work.

### Generation of HSPB6-Overexpressing bladder cancer cells

The vectors for lentiviral-mediated overexpression, including both the control and HSPB6-specific lentiviruses, were supplied by GeneChem, located in Shanghai, China. T24 and RT-112 bladder cancer cell lines underwent transfection with these plasmids using the GP-transfect-Mate system. Following transfection, cells were also subjected to lentiviral infection with the aid of polybrene to enhance virus entry. Subsequently, the selection of successfully infected cells was conducted using puromycin to isolate those with HSPB6 overexpression.

### Lentivirus transduction

To generate stable cell lines with HSPB6 knockdown and overexpression, as well as TCF7L1 overexpression, we performed stable infections using lentiviral plasmids containing HSPB6 small hairpin RNA (shRNA) sequences (as provided in Table S1), the coding sequence (CDS) of HSPB6, and the CDS of TCF7L1 (Genechem, Shanghai, China). 1.5 × 105 tumor cells were placed in 24-well plates and cultured overnight in 500 μl medium at 37 °C. Then, when the cell density was 30%-50%, the lentiviral titer was calculated according to MOI = 10 and infected with T24 cells. After 24 h, the cell medium was removed and 500 μl fresh medium was replaced to continue cell culture. The infection rate was examined by fluorescence microscope (Olympus) 48–72 h later. Stable cell lines were screened with puromycin (Solarbio) for 3 to 4 weeks, and the expression of HSPB6 and TCF7L1 was confirmed by RT-qPCR and Western blot for follow-up experiments.

### Total RNA isolation and quantitative real-time PCR (RT-qPCR)

We extracted total RNA from the cellular samples using the TRIzol reagent provided by Invitrogen, USA. This step was followed by the synthesis of cDNA, for which we employed the protocol of the RevertAid First Strand cDNA Synthesis Kit from Fermentas, Canada. For the RT-qPCR analysis, we used the MyiQTM system from Bio-Rad, along with the TAKARA Green Real-time PCR Master Mix from Japan for detection purposes. GAPDH served as the internal control gene throughout the process. To quantify the gene expression levels, we applied the 2-ΔΔCT method. The specific primer sequences used are listed in Table S2.

### Western blot analysis

Cellular proteins were extracted using RIPA buffer from Beyotime, China, which was supplemented with a protease inhibitor cocktail also sourced from Beyotime. After extraction, the proteins underwent separation through SDS-PAGE and were then transferred to PVDF membranes. The membranes were then blocked with 10% milk for a duration of 2 h. Following the blocking step, they were incubated overnight at 4 °C with specific primary antibodies at a dilution ratio of 1:5000, obtained from Abcam, USA. This was followed by incubation with secondary antibodies, compatible with the primary antibodies, at room temperature for 1 h at a dilution of 1:10,000, also from Abcam. The detection of protein bands was achieved using enhanced chemiluminescence (ECL) provided by Tanon. Finally, densitometric analysis of the images was performed using Image J software, version 1.47.

### Evaluation of cell viability using CCK-8 assay

Bladder cancer cells, specifically T24 and RT-112, were plated at a density of 3000 cells per well in 96-well plates. These cells were then allowed to grow over periods of 24, 48, and 72 h. After each specified growth period, we added 10 μl of CCK-8 solution from Yeasen to every well, which then underwent an additional hour of incubation. To evaluate cell viability, we measured the optical density at a wavelength of 450 nm.

### EdU proliferation assay analysis

We assessed the proliferation rates of T24 and RT-112 cells under various conditions using an EdU assay kit supplied by Beyotime, China, adhering strictly to the protocol provided by the manufacturer. Initially, cells were plated in 24-well plates at a density of 100,000 cells per well and were left to attach for 24 h. Following this attachment period, we treated the cells with 10 μM EdU and incubated them for 2 h at 37 °C within an environment containing 5% CO₂. Post-incubation, the cells were fixed using 4% paraformaldehyde from Solarbio and were made permeable with 0.5% Triton X-100, also obtained from Solarbio. After completing the staining process, we determined the percentage of EdU-positive cells by analyzing them with a laser scanning confocal microscope from Olympus.

### Evaluation of cell invasion using transwell assay

The invasive potential of cells was assessed using Transwell chambers from Corning, USA, which were pre-coated with Matrigel, also from Corning. We prepared a suspension of 50,000 cells in 100 μl of serum-free medium and placed it into the upper chamber. The lower chamber was filled with 600 μl of medium containing 10% FBS. This arrangement was incubated at 37 °C within a 5% CO₂ environment for 24 h for migration assessments and 48 h for invasion studies. After the designated incubation times, we stained cells that had migrated to the underside of the membrane with crystal violet for 30 min. To quantify the number of cells that invaded, we counted them in three randomly chosen fields at a magnification of 200x using a brightfield microscope. For quantification, ImageJ software, version 1.47, was employed. To validate the reliability of our findings, we conducted each experiment in triplicate.

### Wound healing assay

To assess cell migration, we cultured cells in 6-well plates until they formed a confluent monolayer. We then created a uniform scratch across this monolayer using the tip of a pipette. After the scratch was made, we placed the cells in a serum-free medium and allowed them to incubate for 24 h, mimicking the wound healing process. We monitored the progress of wound closure by taking pictures at set time intervals and documenting the observations.

### Apoptosis detection

We measured apoptosis levels in T24 and RT-112 cells using the Annexin V-FITC Apoptosis Detection Kit (C1062, Beyotime Institute of Biotechnology), which includes all necessary components for conducting the assay. First, the cells were washed with PBS and then resuspended in 195 μl of Annexin V-FITC binding buffer. Next, they were incubated with 5 μl of Annexin V-FITC and 10 μl of propidium iodide (PI) for 15 min in darkness at room temperature. The analysis of apoptotic cells was performed using a NovoCyte flow cytometer (ACEA Bioscience, Inc.), and the resulting data were analyzed with NovoCyte software (version 1.5.6, ACEA Bioscience, Inc.).

### Cell cycle analysis

Cell cycle progression analysis was performed utilizing a DNA Content Quantitation Assay (Cell Cycle) kit (Beijing Solarbio Science & Technology Co., Ltd), following the provided instructions. Subsequent to transfection and treatment, cells were exposed to 40X RNase A at 37 °C for 20 min, followed by staining with PI solution at 4 °C for 15 min. Distribution of cells across different phases of the cell cycle was evaluated using a flow cytometer.

### Xenograft model establishment

The experimental protocol involving animals received approval from the Experimental Animal Welfare Ethics Committee of Jinzhou Medical University. Subsequently, four-week-old BALB/c-nude mice, acquired from GemPharmatech, were utilized to establish a subcutaneous tumor model. Subcutaneous tumor models were established by subcutaneously injecting 10 × 10⁶ T24 cells into each BALB/c-nude mouse. Upon conclusion of the experiment, humane euthanasia was administered to the mice, and tumor tissues were surgically excised. These tissues underwent weighing, photographic documentation, and preparation for subsequent immunohistochemical examination.

### Immunohistochemistry (IHC) analysis

Tumor samples were fixed in paraffin, and sections were prepared and deparaffinized, followed by rehydration. Antigen retrieval was performed before sections were treated with 3% H2O2 to quench endogenous peroxidases. Sections were blocked using BSA prior to incubation with primary antibodies against HSPB6 and Ki67 overnight at 4 °C. Following the application of secondary antibodies, tissues were subjected to staining with DAB to facilitate visualization. Hematoxylin was employed for nuclear counterstaining.

### Chromatin immunoprecipitation-polymerase chain reaction (ChIP-PCR) assay

T-24 cells underwent fixation with 1% formaldehyde at ambient temperature for a duration of 10 min, followed by termination of the fixation process with 125 mM glycine for 5 min. The cell lysates were subjected to sonication to fragment the DNA. Chromatin fragments contained in 100 μl of these lysates were then precipitated using an antibody specific to TCF7L1 (5 μg; catalog number sc-377519; Santa Cruz Biotechnology, Inc.) or a nonspecific control IgG (5 μg; catalog number 2729; Cell Signaling Technology, Inc.) through an overnight incubation at 4 °C. The resultant immunocomplexes were isolated with A/G-agarose beads (Santa Cruz Biotechnology, Inc.). DNA isolated from these complexes was purified utilizing ChIP DNA clean & concentrator kits (Zymo Research) and subsequently subjected to RT-qPCR analysis, in accordance with previously mentioned protocols.

### Co-immunoprecipitation (Co-IP) assay

After transfection with lentiviral vector for 48h, the cells were lysed with RIPA lysis buffer and centrifuged at 12,000*g* at 4 °C for 10 min. Subsequently, 20 μl Protein A/G PLUS-Agarose (Santa Cruz, CA, USA) was added to every 100 μl supernatant. The primary antibody of HSPB6 and TCF7L1 was turned over and incubated at 4 °C overnight. On the second day, the bound proteins were fully washed with washing buffer eight times, and the corresponding secondary antibody was incubated at 4° overnight. Finally, SDS-PAGE Western blot analysis was performed.

### Dual-luciferase reporter gene assay

We integrated both the TCF7L1 binding motif and the entire HSPB6 promoter into the pGL3-basic vector, provided by Hunan Fenghui Biology. TCF7L1 and HSPB6-promotor target sequence sequencing results are listed in Table S3. T24 cells were plated at a concentration of 100,000 cells per well in 24-well plates. On the next day, the cells were co-transfected with 2 μg of the pGL3-based reporter constructs and 2 μg of the pRL-SV40 vector, using Lipofectamine 3000 from Invitrogen, part of Thermo Fisher Scientific, Inc. Forty-eight hours after the transfection, we measured the Firefly luciferase activity employing the Dual-Luciferase Reporter Assay System by Promega Corporation. This measurement was then normalized to the Renilla luciferase activity.

### Immunofluorescence protocol

After the incubation period, we treated the cells with 4% paraformaldehyde for 20 min to fix them, followed by three PBS washes. The cells were then permeabilized with 0.5% Triton X-100 for 20 min, with subsequent washes in PBS. We blocked the cells at room temperature for 30 min using goat serum. This step was followed by an overnight incubation at 4 °C with an anti-E-cadherin primary antibody at a 1:50 dilution (ABclonal, A3044), then the cells were washed again in PBS. After the incubation with the primary antibody, cells were treated with a fluorescent secondary antibody (goat anti-Rabbit Alexa 594, ZSGB-BIO) diluted at 1:200 and incubated for 2 h in darkness at room temperature. Finally, the cell nuclei were stained with DAPI (Sigma Aldrich) for 2 min in darkness. Cellular imaging was performed using an inverted fluorescent microscope.

### Statistical analysis

Statistical evaluations were conducted utilizing SPSS software version 21.0 (SPSS) along with GraphPad Prism software version 8.0 (GraphPad Software). The data are presented as mean values ± standard deviations (SD). To determine the statistical significance of differences between the two groups, we used the Student's *t* test, considering a *p*-value of less than 0.05 as significant.

## Data availability

The data utilized in this study can be accessed through the TCGA-BLCA cohort from The Cancer Genome Atlas (TCGA) database. Additionally, microarray data produced in this research are available at https://zenodo.org/records/10793623.

## Supporting information

This article contains supporting information.

## Conflict of interest

The authors declare that they have no conflicts of interest with the contents of this article.
