# Supplementary material for: Transcription factor TCF7L1 targeting HSPB6 is involved in EMT and PI3K/AKT/mTOR pathways in bladder cancer
Source: J Biol Chem. 2024 Nov 26;301(1):108024. doi: 10.1016/j.jbc.2024.108024 (PMC11728895; doi:10.1016/j.jbc.2024.108024)
Supplement: Supplementary Figure [file mmc1.docx]

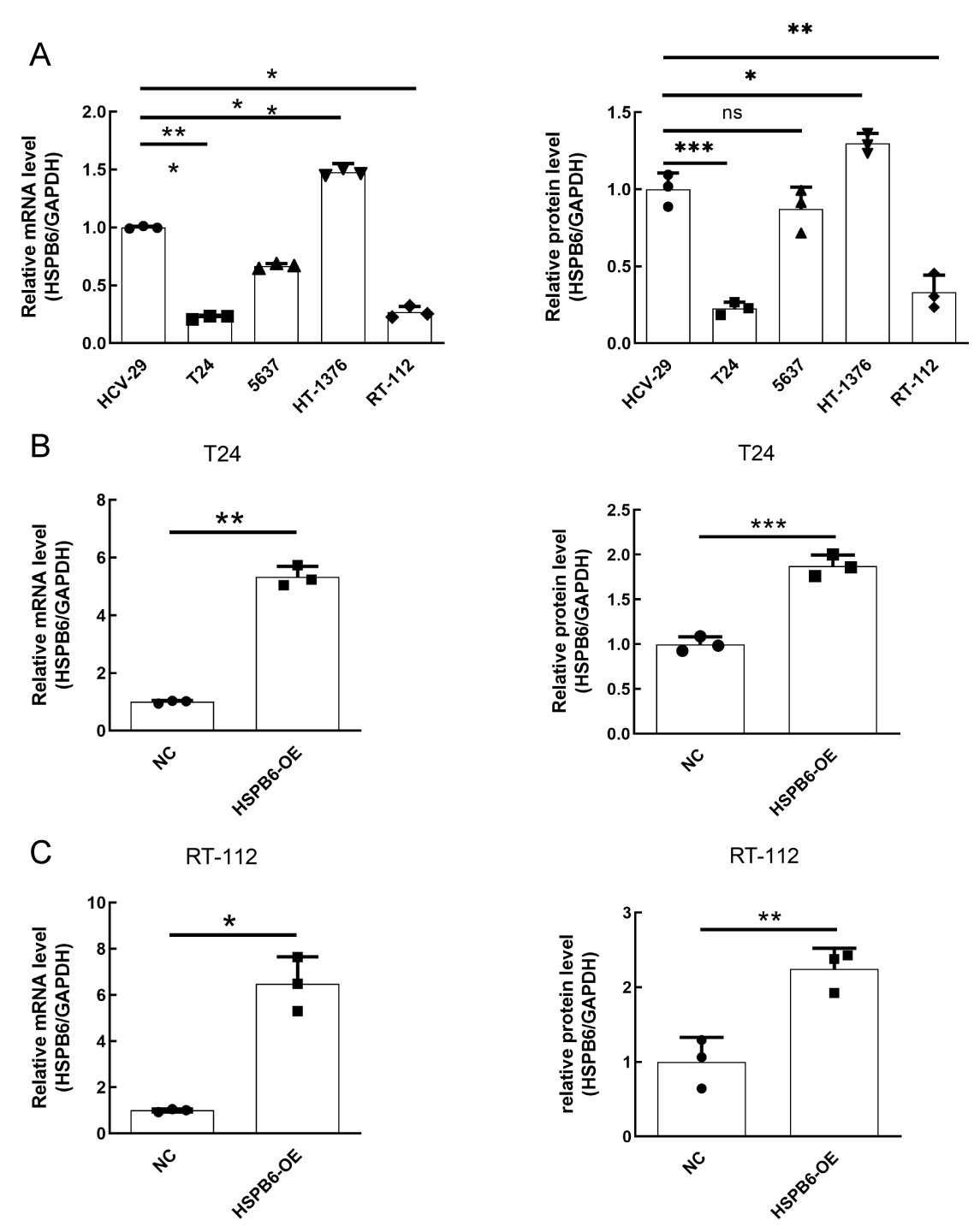


Supplementary Figure S1. Screening bladder cancer cell lines and verifying the efficacy of overexpression of HSPB6 in T24 and RT112 cell lines. **A** The expression level of HSPB6 in multiple bladder cancer cell lines ((5637, HT-1376, T24 and RT-112 cells) and normal bladder cells (HCV-29 cells) was determined using RT‑qPCR. *** P<0.001 vs. HCV-29 cells.  **B, C** qPCR and western blot analysis validated HSPB6 expression in T24 and RT-112 cells after HSPB6 overexpression. **p<0.001, Student’s t-test.


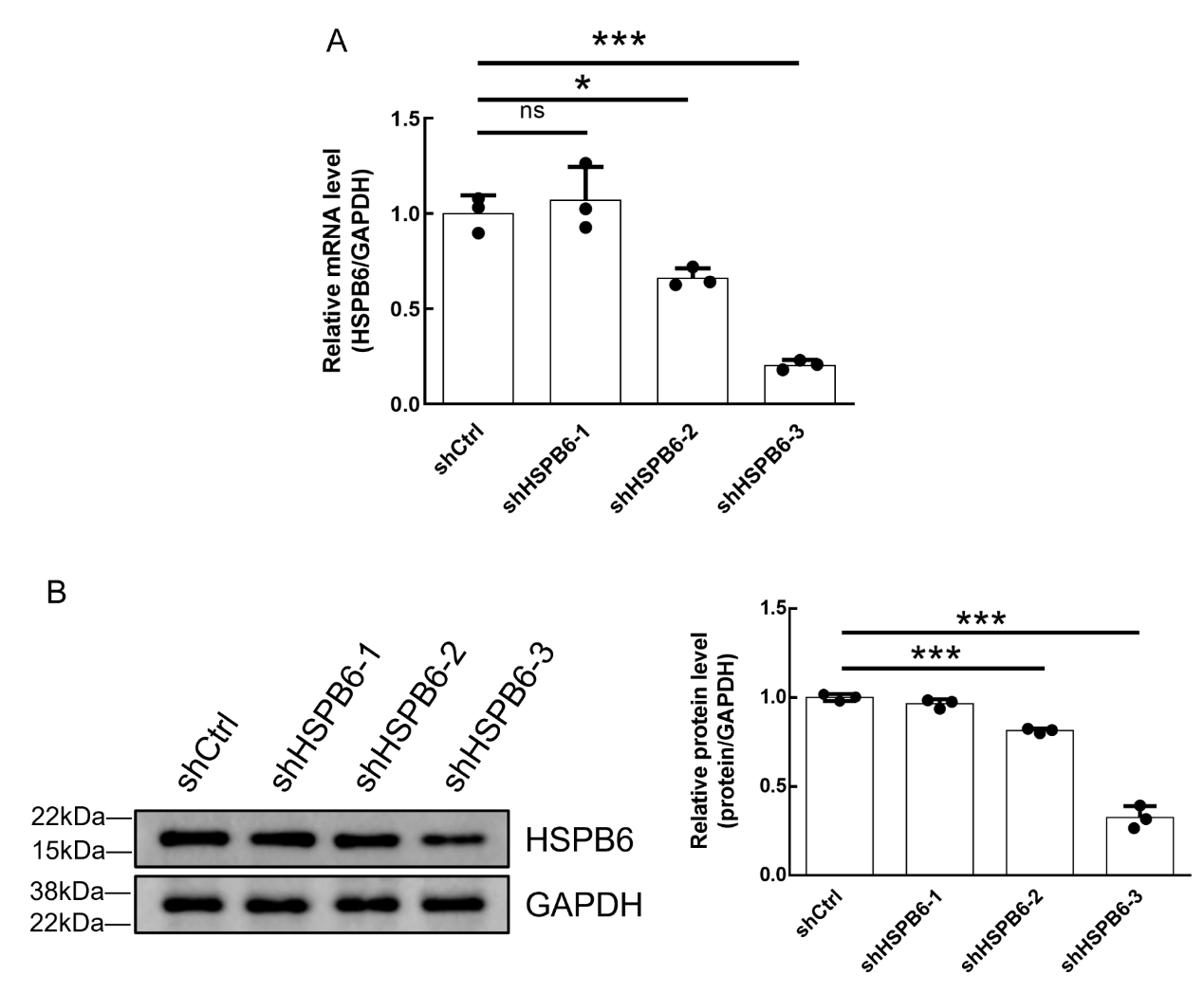


Supplementary Figure S2. 3 different knock down sequences of HSPB6. A The expression level of HSPB6 in control group and knockdown sequence groups using RT‑qPCR. B The protein expression of control group and knockdown sequence group. ***p<0.001; **p<0.01; *p<0.05.
