# Supplementary material for: Transcription factor TCF7L1 targeting HSPB6 is involved in EMT and PI3K/AKT/mTOR pathways in bladder cancer
Source: J Biol Chem. 2024 Nov 26;301(1):108024. doi: 10.1016/j.jbc.2024.108024 (PMC11728895; doi:10.1016/j.jbc.2024.108024)
Supplement: Supplementary Table S1 [file mmc2.docx]

Supplementary Table 1. Sequences of shRNAs targeting HSPB6 and the lentiviral plasmids used for stable cell line generation

| Gene | sequence (5’-3’) |
| --- | --- |
| shRNA-1(HSPB6) | CTGTGCCTGTGCAGCCGTCTTGG |
| shRNA-2(HSPB6) | CCCCACCACGCTCGCCCCCTACT |
| shRNA-3(HSPB6) | CGCCCGGATGAGCACGGATTCGT |
