# Supplementary material for: Transcription factor TCF7L1 targeting HSPB6 is involved in EMT and PI3K/AKT/mTOR pathways in bladder cancer
Source: J Biol Chem. 2024 Nov 26;301(1):108024. doi: 10.1016/j.jbc.2024.108024 (PMC11728895; doi:10.1016/j.jbc.2024.108024)
Supplement: Supplementary Table S2 [file mmc3.docx]

Supplementary Table2. Primers used in this study.

| Gene | Forward primer（5’-3’） | Reverse primer（5’-3’） |
| --- | --- | --- |
| HSPB6 | ATTGCTGTCAAGGTGGTGGGC | GCGGTAGCGACGGTGGAACT |
| GAPDH | ATCATCAGCAATGCCTCC | CATCACGCCACAGTTTCC |
