# Supplementary material for: Transcription factor TCF7L1 targeting HSPB6 is involved in EMT and PI3K/AKT/mTOR pathways in bladder cancer
Source: J Biol Chem. 2024 Nov 26;301(1):108024. doi: 10.1016/j.jbc.2024.108024 (PMC11728895; doi:10.1016/j.jbc.2024.108024)
Supplement: Supplementary Table S3 [file mmc4.docx]

Supplementary Table 3. Sequencing Results of TCF7L1 Binding Motif and HSPB6 Promoter Sequences Integrated into the pGL3-Basic Vector.

**TCF7L1 sequencing results：**

GAGCTCGTTTAGTGAACCGTCAGATCGCCTGGAGACGCCATCCACGCTGTTTTGACCTCCATAGAAGATTCTAGAGCTAGCGCCACCATGCCCCAGCTCGGCGGCGGGGGCGGCGGCGGCGGCGGCGGCAGCGGGGGAGGCGGCGGCTCCAGCGCCGGGGCGGCCGGCGGAGGGGACGACCTCGGGGCGAACGACGAGCTGATCCCCTTCCAGGACGAGGGGGGCGAGGAGCAGGAGCCGAGCAGCGATAGCGCCTCGGCGCAGCGGGACCTAGACGAGGTCAAGTCGTCCCTGGTCAACGAGTCGGAGAACCAGAGCAGCAGCTCGGACTCGGAGGCGGAGAGGCGCCCGCAGCCCGTCCGGGACACTTTCCAGAAGCCGCGGGACTATTTCGCCGAAGTGAGAAGGCCTCAGGACAGCGCGTTCTTTAAAGGACCCCCGTACCCTGGGTACCCCTTCCTGATGATCCCGGACCTGAGCAGCCCGTACCTCTCCAACGGACCCCTGTCTCCCGGAGGAGCGCGCACCTACCTGCAGATGAAATGGCCCCTCCTCGATGTCCCCTCCAGCGCCACAGTCAAGGACACGAGGTCACCATCTCCAGCACACTTGTCTAATAAAGTTCCTGTCGTTCAGCACCCGCATCACATGCATCCGCTGACTCCCCTCATCACCTACAGCAATGACCACTTCTCCCCCGGCTCCCCTCCCACCCACCTCTCCCCAGAGATCGATCCAAAGACAGGAATCCCCCGGCCCCCTCACCCATCCGAGCTGTCACCGTATTACCCACTCTCTCCCGGAGCTGTCGGACAAATCCCCCACCCCCTCGGCTGGCTCGTCCCACAGCAAGGCCAGCCCATGTACTCCCTTCCTCCCGGTGGCTTCCGGCACCCTTACCCCGCCCTCGCCATGAACGCCTCGATGTCCAGCCTGGTCTCCAGTCGGTTCTCTCCTCACATGGTGGCTCCTGCCCACCCTGGCCTGCCCACCTCAGGGATCCCCCACCCTGCCATCGTCTCCCCCATCGTCAAGCAGGAACCGGCACCCCCCAGCCTGAGCCCTGCAGTGAGCGTGAAATCACCAGTCACCGTGAAAAAGGAGGAGGAAAAGAAGCCCCACGTGAAGAAGCCTCTGAATGCCTTCATGTTGTATATGAAGGAGATGAGGGCCAAGGTGGTGGCTGAGTGCACCCTGAAGGAAAGTGCAGCCATTAACCAGATCCTTGGAAGAAAGTGGCACAACCTGTCTCGAGAAGAACAGGCCAAGTACTACGAGCTGGCCCGGAAGGAGCGGCAGCTTCACTCGCAGCTCTACCCAACCTGGTCAGCCCGGGACAACTATGGTAAGAAAAAGAAGAGGAAGAGAGAAAAGCAGCTGTCCCAGACACAGTCACAGCAGCAAGTCCAGGAGGCAGAGGGTGCCCTGGCCTCCAAGAGCAAGAAGCCATGTGTTCAGTACCTGCCCCCCGAGAAGCCCTGTGACAGCCCTGCCTCCTCCCACGGGAGCATGCTGGACTCCCCGGCCACTCCCTCTGCAGCTTTGGCCTCACCAGCTGCCCCTGCTGCCACCCATTCGGAGCAAGCCCAGCCCCTCTCCCTCACCACCAAACCAGAAACCCGGGCCCAGCTGGCTCTCCACTCTGCCGCCTTCCTGTCGGCTAAGGCTGCAGCCTCCTCCTCTGGGCAGATGGGCAGCCAGCCTCCCCTCCTGTCCCGGCCCCTCCCCCTTGGGTCCATGCCCACAGCTCTGCTGGCCTCTCCCCCGTCCTTCCCCGCCACGCTCCATGCCCACCAGGCCCTCCCGGTGCTACAGGCCCAGCCTCTTTCCCTGGTCACCAAGTCTGCCCACGGAATTCTCGACTCTAGAGGATCCCCGGGTACCGGTATGGACTACAAGGATGACGATGACAAGGATTACAAAGACGACGATGATAAGGACTATAAGGATGATGACGACAAATGAGCTAGTCCGCGGCCGCGAAGGATCTGCGATCGCTCCGGTGCCCGTCAGTGGGCAGAGCGCACATCGCCCACAGTCCCCGAGAAGTTGGGGGGAGGGGTCGGCAATTGAACGGGTGCC

**HSPB6-promotor sequencing results：**

CTCGTTTAGTGAACCGTCAGATCGCCTGGAGACGCCATCCACGCTGTTTTGACCTCCATAGAAGATTCTAGAGCTAGCGCCACCTATTATACTTACATTGTTGCACAACTTGTAATTTTTAAACTAGTTGTGAACCTTTTCCTTCTTAGCAAATATTCATCTAATTACCACTATTTATTGTTATTATTATTATTTGAGACAGGATCTTTCTCTGCTGCCCAGGCTGCAGTGCAGTGGAGATCACAGATGACTGCAACCTTGAAGTCATAGACTCAAGTGATTCTCGTGCCTCAGCCTTCCAAGTAGCTGGGACTACAGGCATGTGCTACCACACACAGCTAATTTTTAAATTTCTTTTGTAGAGACAAGGGTCTCACTATGTTGCTCAGGCTGGTCTGGAACTCCTGGCCTCAAGCAATCCTGCCTCGGCCTCCCAAAGTGCTGGGATTCCAGGGATGAGCCACCATGCTAGGCTCTAAATTATAATTATTAATGGCTATGTATTTTTCTTATTATGAATGCATCTTTTTTTTTTTTTTTTTTTTTTTTTGAGATGGAGTCTCGCACTGTCACCCCAGCTGGAGCGCAGTGGCGCGATCTCGGCTCACTGCAACCTCCGCCTGCTGGCTTCAAGGGATTTTCCTATCTCAGCCTCCTGAGTAGCTGGCATTACAGGCACCTGCCACCACGCCTACCTTTTTTTTTTTTTTTTTGTATTTTTAGTAAAGACGGGGTTTCACTATGTTGGTCAAGCTGGTCTCAAACTCCTGACCTCATGATCCGCCCGCCTCGGCCTCCCAAAGTGCTGGGATTACAGGTGTGAGCCACCGCGCCCAAACGAATGCATCATATTTTATTGGACTCCTACTTTGAAATATTTAGGAATTTTCAGTGTTTACTATGACAAATAGCACTGTGATTAAAAAAAAATGTAGTCAAATCCACTTTTGCTGTTGATTTTTTTTTTTTTAATCTGTAGAGACATCGTCTCGCTATGTTGCCCAGGCTGGTCTGGAACTCCTGGCCTCAAGCGATCCTCCTGCTTTGGCCTCCCAAAGTGCGTGGGCCACCGTGACCAGCAGTTATTTATTTTCTTTGGTTGTGTGTGTGTGTGTGTGTGTGTGTGTGTGTGTGTGTGTGTATGTATGTATTGAGACGGAGCTTGCTCTGTTGCCCAGGCTAGAGTGCAGTGGCACGATCTCGGTGCATGCAACCTTCACCTCCCGGGTTCAGGCGATACTCGTGCCTCAGCCTCCCGAGTATCTGGGATTACAGGCGTGAGCCACCATGCCCAGCTAATTTTTGTATTTTCAGTAGAGACGGGGGGTGCGGGTGGGGGAATTTCACCATGTTGACCAGGCTGGTCTCGACTCCTGACCTACGGTGATCCGCCTGCCTCGGTCTCCCAAAGTGCTGGAATTATGGGCGTGAGCCACCGCGCCGGGCCAGCTATTGATTTTTTGAAAGGATTTTTAAAAAATAATGTTGCTTTGAAAATTTTTATTTATGACTAGCTCTTTTGGTTAAAACTTGGCTATGGTTTCTCTGGAGTGATCCTGAGTTGGATAATTTTGGGAAAGTTAGAGAATCTAATTAAAAACATTTTCAAATGCTTATTAATTGTAACAAAAGAGATATCACAGGATCCATAAAGAGCGCTAGCCCGAAACGTCTTTTGATGCAGTAATTCCTGGGCACCGGCATCGCTCACATTCTCCTCTCAGAATTACACCGGGAGGGGTCGCGAACCTCTGCTAGCCGCTCTCGCTGCGATCCGGAGATACCCAGACACCGGACTCCTTGAGTTCACAGCCCGGATGCAGAAGGGAATCCTAGCAGCGCCGTACCGGATGTGTGAGTGGAAGTGACTGAGGTGAACTCACGACCGATTGGTTCTCCCGAGTGGACCCAAGCCTGTTTTCACCTGCCACTGGCCGCGTGGTTTGTCTATTTGAACAGTCCCCGCCCCCTGGAGAAGCCGGGTCCTGAAGCTTCTCTTCTTCATTACTATTGGCCTGTTTTTCAGTCCATCTTCTCCCTATACCTGTCGATTGGTCCTCAGGGCTGGGAACCCTCCTTCCCTCGGAATTCTCGACTCTAGAGGATCCCCGGGTACCGGTATGGACTACAAGGATGACGATGACAAGGATTACAAAGACGACGATGATAAGGACTATAAGGATGATGACGACAAATGAGCTAGTCCGCGGCCGCGAAGGATCTGCGATCGCTCCGGTGCCCGTCAGTGGGCAGAGCGCACATCGCCCACAGTCCCCGAGAAGTTGGGGGGAGGGGTCGGCAATTGAACGGG

## Note: The red part is the sequencing result of the target sequence.
